# The characteristics and phylogenetic relationship of two complete mitochondrial genomes of *Cottus pollux* (scorpaeniformes: cottidae)

**DOI:** 10.1080/23802359.2023.2301014

**Published:** 2024-01-08

**Authors:** Bong Han Yun, Yong Hwi Kim, Ho-Seop Han, In-Chul Bang

**Affiliations:** Department of Biology, Soonchunhyang University, Asan, Republic of Korea

**Keywords:** Sculpin, Cottidae, *Cottus pollux*, mitogenome, phylogenetic analysis

## Abstract

We report two complete mitochondrial genomes of *Cottus pollux* based on specimens collected from Deokdong and Hoam Streams in the Republic of Korea. The two complete mitochondrial genomes were 16,558 and 16,557 bp long. Both contain the 37 standard genes (13 protein-coding genes, 22 transfer RNA genes, two ribosomal RNA genes, and one control region) in the same order and have similar nucleotide compositions. According to the phylogenetic tree constructed using the maximum-likelihood method, *C. pollux* is closely related to *C. reinii*. The genetic information provided by the complete mitochondrial genome of *C. pollux* will contribute to an understanding of the phylogenetic position, evolutionary relationships, and biogeographical patterns of this species within the genus *Cottus*.

## Introduction

*Cottus pollux* Günther, 1873 had been considered endemic to Japan, where it is found mainly in Honshu and northwest Kyushu (Hosoya 2015), as well as southern Far East Asia. However, morphological and molecular phylogenetic analyses in a recent study by Yun et al. (2022) of *Cottus* fish populations in the Hyeongsan River and Daejong Stream basins (southeastern Korean Peninsula) in the Republic of Korea revealed them to be *C. pollux* rather than *C. koreanus*, as previously thought (Byeon and Lee 2017). In addition to this species, striking similarities in fauna have been reported between the southeastern Korean Peninsula and the western Japanese Archipelago (Kyushu and western Honshu) due to the connectivity of the two regions until approximately 25 MYA and periodic opening and closure of the Korea Strait during the Pleistocene (Chae 1999; Ohnishi et al. 2009; Wakita 2013; Taniguchi et al. 2021).

*Cottus pollux* has been found in a very narrow range only in the uppermost reaches of the Hyeongsan River and Daejong Stream basins, which flow into the southern part of the East Sea (Sea of Japan) from the Republic of Korea (Byeon and Lee 2017; Yun et al. 2022). The populations in these basins have declined due to frequent typhoon damage and river construction (Yoon and Kim 2004). To conserve species threatened with extinction, it is important to preserve genetic resources from native species (Lakra et al. 2007; Kim et al. 2022). Here, we determined the complete mitochondrial genomes of *C. pollux* currently occurring in two watersheds in the Republic of Korea. These mitogenomes will expand the available genetic data on *C. pollux* and provide information for species identification and evolutionary, phylogenetic, and biogeographical studies of the genus *Cottus*.

## Materials and methods

### Sample collection

**Figure 1.**
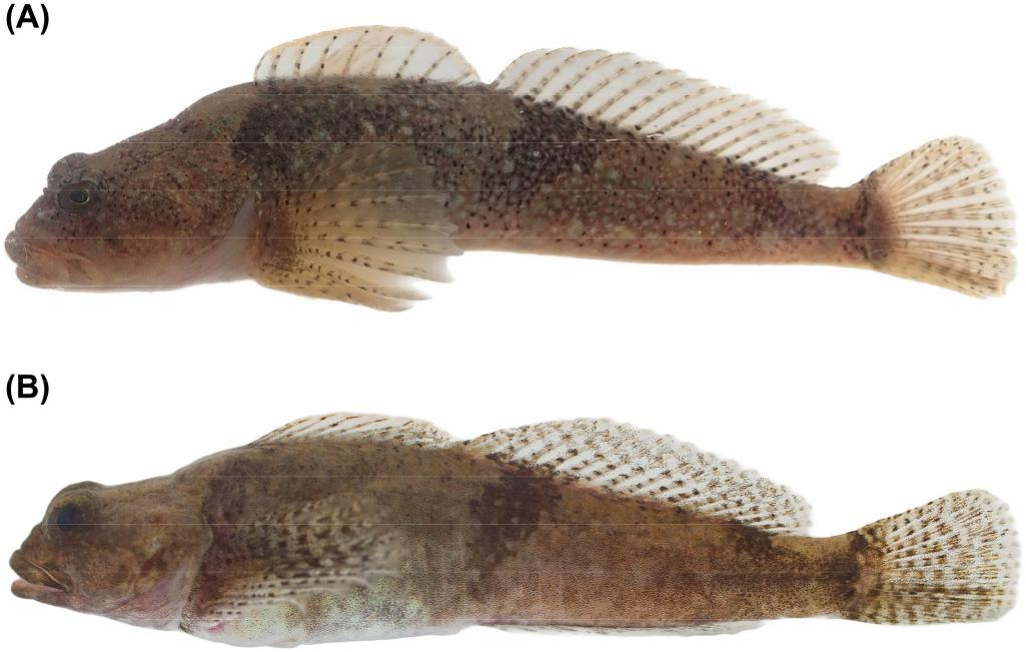
The specimens of *Cottus pollux* from Deokdong (A) and Hoam (B) Streams in the Republic of Korea (photos by the authors). Main diagnostic characters: absence of palatine teeth, 12–14 unbranched pectoral fin rays, absence of bands or spots on the pelvic fin, and absence of blackish spots on the head.

One of the *C. pollux* specimens (voucher no. SUC25238; Figure 1(A)) was collected on 14 October 2021 from Deokdong Stream (DDS), a tributary of the Hyeongsan River, Republic of Korea (35°52′34.49″N, 129°19′34.47″E). The other (SUC26332; Figure 1(B)) was collected on 2 April 2022 from Hoam Stream (HAS), a tributary of the Daejong Stream, Republic of Korea (35°49′59.18″N, 129°24′19.32″E). The specimens were fixed in 99.9% ethanol and kept in the specimen storage facility of Soonchunhyang University (Prof. In-Chul Bang: incbang@gmail.com).

### DNA extraction, sequencing, assembly, and annotation

Genomic DNA (gDNA) was extracted from the pectoral fin using a HiGene™ Genomic DNA Prep Kit (BIOFACT, Daejeon, Republic of Korea), and a genomic library for next-generation sequencing (NGS) was prepared with extracted gDNA using the MGIEasy DNA Library Prep Kit (MGI Tech, Shenzhen, China). NGS raw data were produced using the MGISEQ-2000 platform (MGI Tech, Shenzhen, China) with 150 bp paired-end reads, and a contig sequence was assembled using the default options in the *de novo* assembler of the CLC Genomics Workbench 20.04 (CLC, Aarhus, Denmark). A circular sequence was mapped with the filtered datasets using Geneious R11 (Kearse et al. 2012) to verify the complete mitochondrial genome sequence of *C. pollux*, and the final sequence was annotated with the aid of the MITOS web server, a free web server for the annotation of metazoan mitochondrial genomes (Bernt et al. 2013).

### Phylogenetic analysis

A phylogenetic analysis was performed based on the 13 protein-coding genes (PCGs) of *Cottus* species; the sequences were aligned using MUSCLE 3.8.31 (Edgar 2004). The phylogenetic tree was built in RAxML 8.2.10 (Stamatakis 2014) using the maximum-likelihood method, with 1,000 bootstrap replications applied. The partitioning scheme and substitution model (GTR + I + G) were selected according to the corrected Akaike information criterion, using PartitionFinder 2.1.1 (Lanfear et al. 2017).

## Results

### Mitochondrial genome features

**Figure 2.**
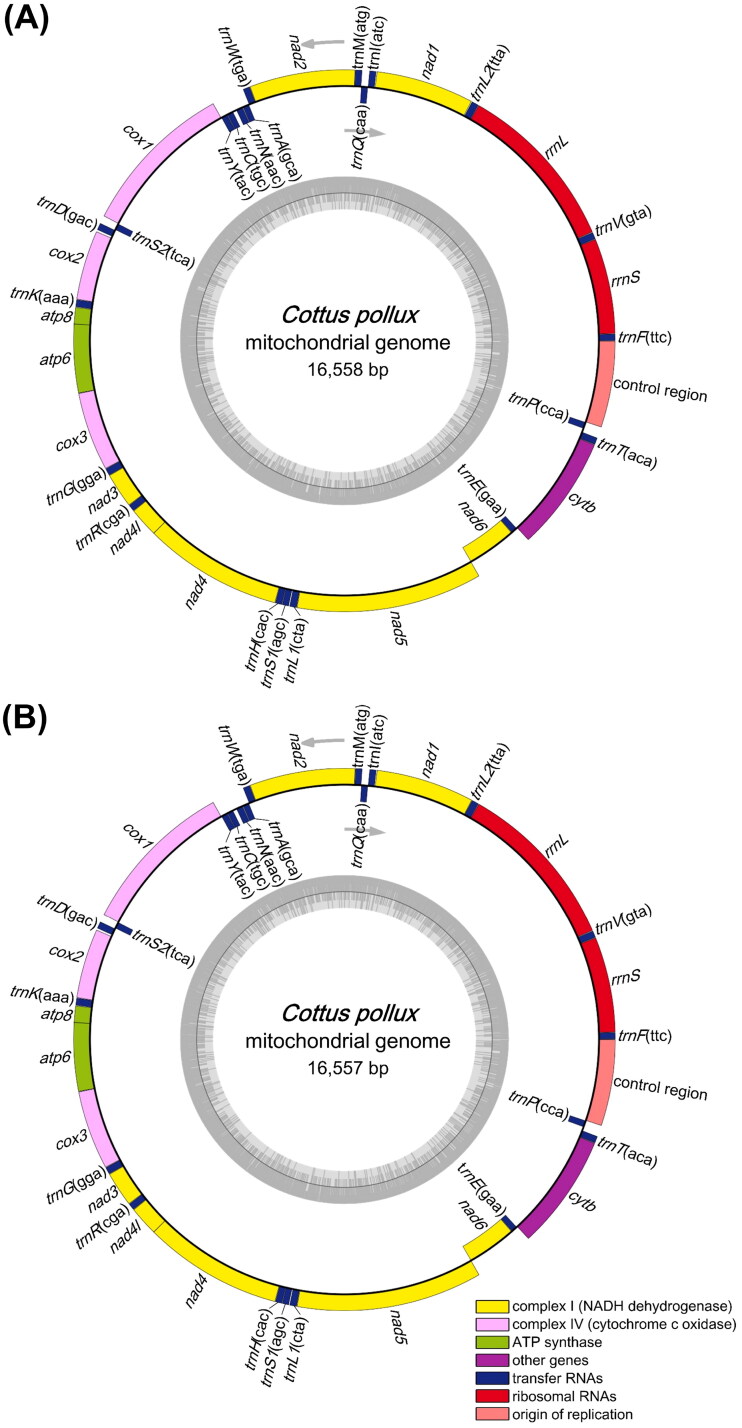
Complete mitochondrial genome pattern maps of *Cottus pollux* from Deokdong (A) and Hoam (B) Streams in the Republic of Korea.

The lengths of the complete mitochondrial genomes of the DDS and HAS specimens were 16,558 bp (GenBank acc. no. ON184012) and 16,557 bp (OR421279), respectively. Both of their mitogenomes included 13 PCGs, 22 transfer RNA (tRNA) genes, two ribosomal RNA (rRNA) genes, and one control region (CR) (Figure 2). The overall base composition of the two mitogenomes was similar, and the A + T contents of the DDS and HAS specimens were 52.50% and 52.46%, respectively. The 12S (945 bp) and 16S (1,686 bp) rRNA genes were located between *tRNA^Phe^* and *tRNA^Val^* and between *tRNA^Val^* and *tRNA^Leu^*, respectively. For 12 of the 13 PCGs, the start codon was ATG; that of the remaining PCG (*cox1*) was GTG. Seven of the PCGs had complete stop codons (TAA or TAG), whereas six terminated with an incomplete stop codon, either T (*cox2*, *nad3*, *nad4*, and *cytb*) or TA (*nad2* and *cox3*). The CR (855 bp) was situated between *tRNA^Pro^* and *tRNA^Phe^*.

### Phylogenetic analysis

**Figure 3.**
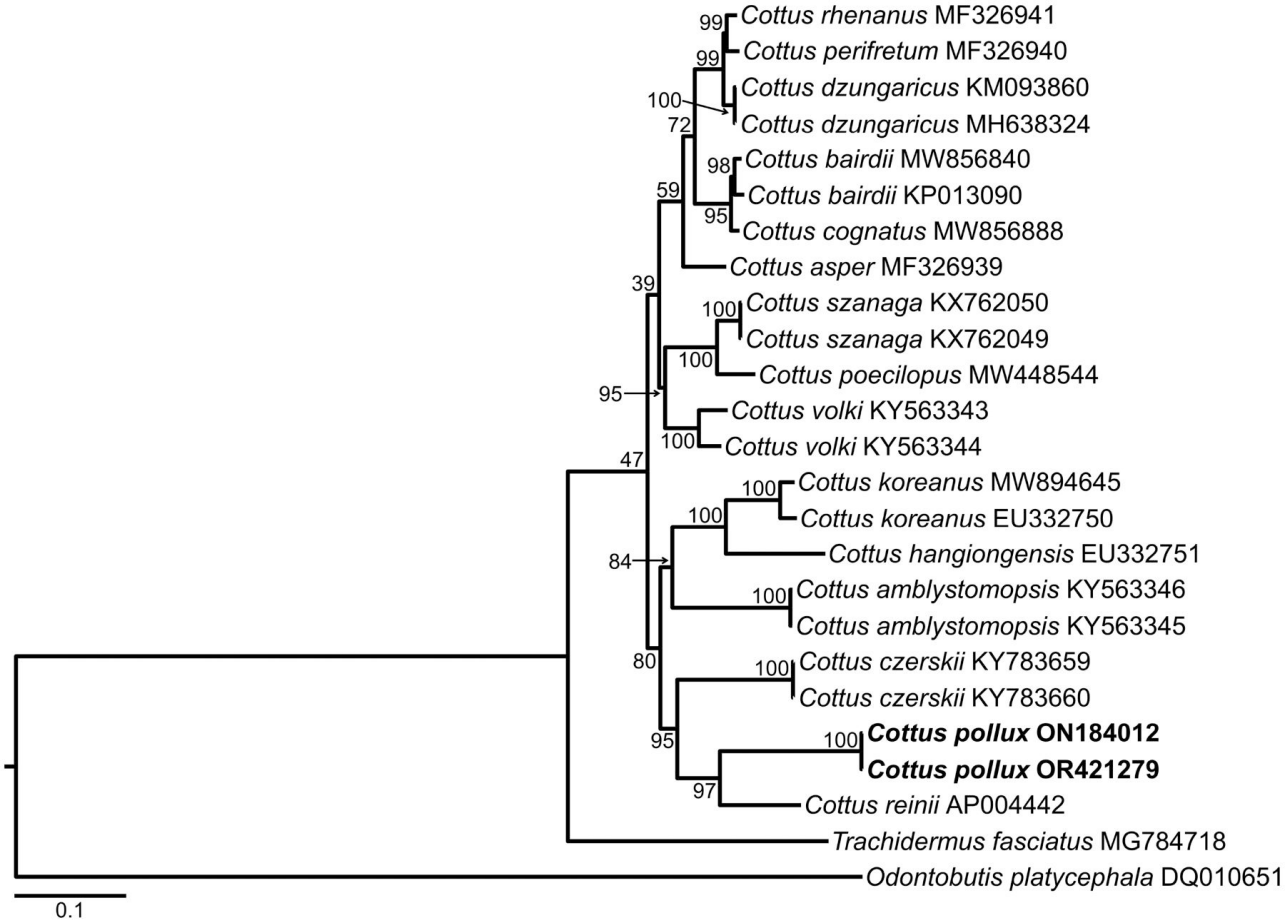
Phylogenetic tree of *Cottus* species produced based on a maximum-likelihood analysis of the 13 protein-coding genes. Bootstrap values are shown at the base of each branch node. The GenBank accession numbers of the species are given after the scientific names, and are as follows: *C. asper* (MF326939; Fast et al. 2017), *C. amblystomopsis* (KY563345, KY563346; Balakirev et al. 2017a), *C. bairdii* (KP013090, MW856840; unpublished), *C. cognatus* (MW856888; unpublished), *C. czerskii* (KY783659, KY783660; Balakirev et al. 2017b), *C. dzungaricus* (KM093860; Ao et al. 2016) (MH638324; Yang et al. 2019), *C. hangiongensis* (EU332751; Hwang et al. 2013a), *C. koreanus* (EU332750; Hwang et al. 2013b) (MW894645; unpublished), *C. perifretum* (MF326940; Fast et al. 2017), *C. poecilopus* (MW448544; unpublished), *C. pollux* (ON184012, OR421279; this study), *C. reinii* (AP004442; Miya et al. 2003), *C. rhenanus* (MF326941; Fast et al. 2017), *C. szanaga* (KX762049, KX762050; Balakirev et al. 2016), *C. volki* (KY563343, KY563344; Balakirev et al. 2017c), *trachidermus fasciatus* (outgroup; MG784718; Zhu et al. 2018), *odontobutis platycephala* (outgroup; DQ010651; Ki et al. 2008).

In the phylogenetic tree, the *C. pollux* specimens collected from the two locations (DDS and HAS) formed a clade with high statistical support (bootstrap values = 97) with *Cottus reinii*, which is endemic to Japan (Hosoya 2015) (Figure 3).

## Discussion and conclusion

This study is the first to characterize the complete mitochondrial genomes of *Cottus pollux* collected from two locations (DDS and HAS) in the Republic of Korea. The size, composition, and gene arrangement of the complete mitochondrial genomes were similar to those reported for other *Cottus* species (Hwang et al. 2013a, 2013b; Balakirev et al. 2016, 2017a, 2017b, 2017c; Fast et al. 2017). According to the phylogenetic analysis, *C. pollux* was most closely related to *C. reinii. C. pollux* and *C. reinii* have been reported as sister relationship that are morphologically similar but differ in aspects of their life histories (fluvial vs. both amphidromous and lacustrine). Their close relationship was confirmed in molecular phylogenetic studies using *atp8*/*6*, *cytb*, and the CR (Yokoyama and Goto 2005; Goto et al. 2015; Dolganov and Saveliev 2022). Yun et al. (2022) supported the species identification of Korean and Japanese *C. pollux* based on morphological analysis and their formation of a clade in the phylogenetic tree of *Cottus* species based on nuclear *ITS1* and mitochondrial *cytb*. The complete mitogenome of Japanese *C. pollux* has not been reported, so it was excluded from the phylogenetic tree in the current study, but a comprehensive whole genome-based phylogenetic analysis including it is needed in the future.

In conclusion, the genomic data of *C. pollux* reported herein will provide a solid basis for understanding the phylogenetic position, evolutionary relationships, and biogeographical patterns of this species within the genus *Cottus*.

## Supplementary Material

Supplemental Material

## Data Availability

The genome sequence data that support the findings of this study are openly available in GenBank of NCBI at [https://www.ncbi.nlm.nih.gov] (https://www.ncbi.nlm.nih.gov/) under the accession no. ON184012 and OR421279. The associated BioProject, SRA, and Bio-Sample numbers are PRJNA932022, SRR18668187, SRR25732683, SAMN33142986, and SAMN37129716, respectively.
