# Supplementary material for: The characteristics and phylogenetic relationship of two complete mitochondrial genomes of Cottus pollux (scorpaeniformes: cottidae)
Source: Mitochondrial DNA B Resour. 2024 Jan 8;9(1):55–9. doi: 10.1080/23802359.2023.2301014 (PMC10776038; doi:10.1080/23802359.2023.2301014)

**Supplemental material**

Figure S1. Graphs showing the read mapping depth of the complete mitochondrial genome sequences of *Cottus* *pollux* from Deokdong (A) and Hoam (B) Streams in the Republic of Korea using Geneious software. The read mapping depth is represented by the blue scale bar. The x-axis and y-axis indicate nucleotide position and coverage, respectively.
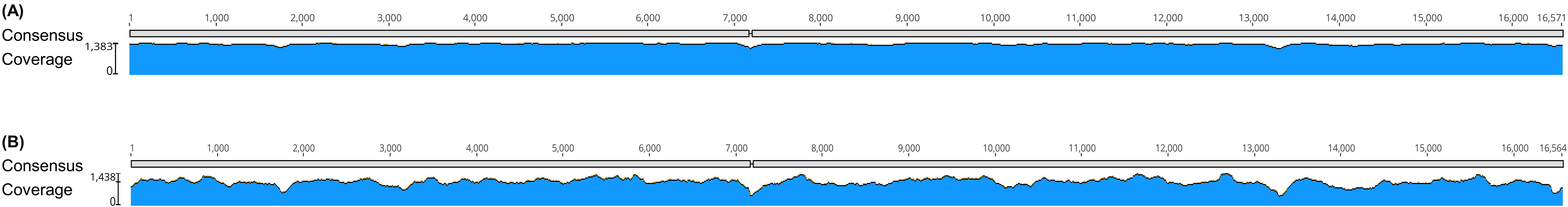

Supplement: Supplemental Material [file TMDN_A_2301014_SM1866.doc]
